# Deep learning strategy for small dataset from atomic force microscopy mechano-imaging on macrophages phenotypes

**DOI:** 10.3389/fbioe.2023.1259979

**Published:** 2023-10-04

**Authors:** Hao Wu, Lei Zhang, Banglei Zhao, Wenjie Yang, Massimiliano Galluzzi

**Affiliations:** ^1^ School of Management Science and Engineering, Anhui University of Finance and Economics, Bengbu, Anhui, China; ^2^ Shenzhen Institute of Advanced Technology, Chinese Academy of Science, Shenzhen, Guangdong, China

**Keywords:** deep learning, small dataset, atomic force microscopy, mechano-imaging, macrophages

## Abstract

The cytoskeleton is involved during movement, shaping, resilience, and functionality in immune system cells. Biomarkers such as elasticity and adhesion can be promising alternatives to detect the status of cells upon phenotype activation in correlation with functionality. For instance, professional immune cells such as macrophages undergo phenotype functional polarization, and their biomechanical behaviors can be used as indicators for early diagnostics. For this purpose, combining the biomechanical sensitivity of atomic force microscopy (AFM) with the automation and performance of a deep neural network (DNN) is a promising strategy to distinguish and classify different activation states. To resolve the issue of small datasets in AFM-typical experiments, nanomechanical maps were divided into pixels with additional localization data. On such an enlarged dataset, a DNN was trained by multimodal fusion, and the prediction was obtained by voting classification. Without using conventional biomarkers, our algorithm demonstrated high performance in predicting the phenotype of macrophages. Moreover, permutation feature importance was employed to interpret the results and unveil the importance of different biophysical properties and, in turn, correlated this with the local density of the cytoskeleton. While our results were demonstrated on the RAW264.7 model cell line, we expect that our methodology could be opportunely customized and applied to distinguish different cell systems and correlate feature importance with biophysical properties to unveil innovative markers for diagnostics.

## 1 Introduction

Macrophages are involved in every stage of the acute immune response as well as in the regulation of tissue homeostasis and in the process of tissue repair. As professional phagocytes, they detect, engulf, and digest particles, microbes, and apoptotic cell debris. When an individual is healthy, the equilibrium of different activation phases (phenotypes) is regulated in order to promote inflammation during the pathogens’ neutralization and regeneration after the resolution of infection. Dysregulation of this equilibrium is involved in many auto-immune or inflammatory diseases. Therefore, for diagnostics and treatment, the ability to recognize and detect different phenotypes is very important and usually performed with staining agents that can influence cell receptors and modify the cells’ status. In this context, we wish to explore biomechanics as a source of innovative markers to distinguish the macrophages’ phenotypes.

Cell mechanics is related to cellular response to the mechanical forces exerted by the cell’s microenvironment, including neighboring cells and the extracellular matrix. The ability of cells to deform and change upon mechanical stress is critical for homeostasis and all dynamic processes in tissues and organs (Li et al., 2018). Mechanical deformability is supporting the complex, dynamic, and anisotropic nature of cells, which must respond in both space and time to the chemical-physical cues presented by the cellular microenvironment. Changes in the mechanical properties of cells often correlate with different cell types and disease states such as cancer (Cross et al., 2007; Suresh, 2007) but are less investigated in immunology.

Atomic force microscopy (AFM) is one of the best techniques to directly access the mechanical property of macrophages correlated with the structural organization of cytoskeletons. Until now, only a few AFM studies have been performed on macrophages, (Rotsch et al., 1997; Leporatti et al., 2006; Roduit et al., 2012), and especially phenotype activation was investigated on fixed hardened cells (Pi et al., 2014).

The main challenge in this work is to exploit AFM nanomechanics (quantified as Young’s modulus and adhesion) as additional dimensions to improve the accuracy in AI-based imaging classification. In this framework, one of the best methods to extract useful information from big datasets is definitely Deep Neural Networks (DNNs), thanks to their performance (using parallel computing), to achieve classification (Bengio and Delalleau, 2011). Deep learning has been tremendously successful in a variety of applications for its strong fitting and predicting ability since 2006 (Hinton and Salakhutdinov, 2006). The use of DNNs is expected to achieve more reliable mechanical biomarkers and deliver classification results for diagnostics with high speed and precision.

Until now there are only a few available applications of AFM mechano-imaging diagnostics aided actively by AI, a technique predicted to have enormous impact for healthcare (Garcia, 2020). For example, Darling and Guilak (2008) applied DNN algorithms on cell nanomechanics from AFM force spectroscopy events (no imaging), analyzing cells populations using a single parameter per cell. While an accuracy of 96% was achieved in distinguishing sub-populations of mesenchymal cell types (different mechanical properties), the method was less performant in distinguishing chondrosarcoma cell lines (similar average elasticity), leading to lower accuracy. The approach of applying DNN on single AFM force curves was also applied to recognize brain cancer tissue (Minelli et al., 2017; Ciasca et al., 2019). Although single force curves showed a certain feasibility during machine learning classification, spatially resolved mechanical properties in a form of mechanical maps are expected to deliver information about properties distribution to be used as important “feature” in object recognition.

Sokolov et al. (2018) delivered one of the first applications of DNNs in biological AFM in order to classify bladder cancer cells. In contrast to the standard analyses, they applied DNNs after extracting quantitative sets of surface parameters from height images (e.g., roughness, directionality, fractal properties). The use of these parameters instead of images substantially decreases the dimension of the data space and the need for large datasets. AFM morphological maps were used to distinguish between neuronal cell development, (Lohrer et al., 2020) showing higher performance than scanning electron microscopy to determine the maturation status of dendritic cells automatically. Their approach is interesting but applies only to morphology, while biomechanics is left unexplored. Recently, Wang et al. (2021) used AFM mechanical maps to train a malignancy classifier through machine learning applied to different cancer lines. Validation of cells with different degrees of morphological and elastic heterogeneity and malignancy showed the good performance of the mechanomics biomarker and its advantage over conventional morphological cytology.

AFM mechanical measurements have inherent drawbacks related to poor automation and low speed, making AFM-based technique less competitive over traditional AI optical imaging diagnostics. In fact, as a data-driven method, deep learning requires large training datasets (usually larger than 10^3^). Although deep learning methods were applied to improve automated cell recognition by AFM, (Rade et al., 2022) it is unlikely that 10^3^ AFM scan images are obtained within a reasonable time and cost. Therefore, use of deep learning on such small datasets will inevitably lead to overfitting, resulting in poor models with high training/verification accuracy but low-test predicting accuracy.

A typical approach to solve this issue is data augmentation. For example, in convolutional neural networks (CNNs), images can undergo a spatial shift, rotations, and flip to generate sets of data invariant from rototranslations (Azuri et al., 2021). The most promising feature of CNNs in classifying images resides in the possibility to extract details, learn, and build a model from input images that can be used to classify new images. However, on small datasets, the performance of the CNN model is limited, and too many morphological details increase uncertainty in classification. The considerable shape diversity within the same phenotype group also makes the classification difficult, especially on small and low-resolution image datasets of living cells (such as most mechano-imaging results). Another typical solution is to pre-train a DNN on a large auxiliary dataset, followed by fine-tuning on the small dataset of interest. However, as in most of the experimental problems, no suitable auxiliary dataset exists in atomic force microscopy mechano-imaging of macrophages. Therefore, a new methodology and protocol must be developed to fully exploit the wealth of information from single AFM images of macrophages’ phenotypes.

The main challenge in this work is to exploit cell nanomechanics by AFM (quantified as Young’s modulus, adhesion, etc.) as additional dimensions to improve the accuracy for automated AI-based imaging classification. In this work we design and demonstrate a general route to train a deep learning model on small AFM datasets based on a multimodal fusion and voting mechanism. The new strategy was based on considering image pixels (correspond of AFM force curves) with spatial attributes to enlarge datasets while maintaining useable information of multi-properties distribution in space and filter out the interference information. On our small training dataset (100 AFM images of RAW264.7 murine macrophages), the predicting classification accuracies for resting, pro-inflammatory, and pro-healing phenotypes reached very high classification accuracies up to 88.9%, 100%, and 100%, respectively. This methodology is expected to obtain high accuracy for other cells’ systems as well while delivering important information on the correlative properties with biological interest.

## 2 Materials and methods

### 2.1 Materials and cell culture

The cell line RAW 264.7 was purchased from Cell Bank of ATCC and stored with dimethyl sulfoxide (DMSO, Sigma-Aldrich) in a frozen pipe in liquid nitrogen for long-term storage. Before experiments, the cells were cultured in Dulbecco’s modified eagle medium (DMEM, Gibco) supplemented with 10% fetal bovine serum (FBS; Sigma-Aldrich), 100 units cm^−3^ penicillin, and 100 units cm^−3^ streptomycin in a 5% CO2 and 98% air-humidified incubator at 37°C. Subcultures were prepared by scraping after washing twice with PBS. Subcultures or culture medium exchanges were routinely established every 3 days. In particular we tested resting phenotype (standard control) and polarized to pro-imflammatory and pro-healing phenotypes after LPS (1 μg cm^−3^ after 24 h) and IL-4 (0.1 μg cm^−3^ after 24 h) stimulation, respectively. Lipopolysaccharide (LPS) is generally found on bacteria surface therefore, it represents the most used choice in immunology to activate the pro-inflammatory phenotype in macrophages. Interleukin 4 (IL-4) is a cytokine released after the inflammation phase to switch pro-inflammatory phenotypes to pro-healing and start the regeneration process. In this work we will use the simplified notation M0 for resting phenotype, M1 for pro-inflammatory, and M2 for pro-healing phenotypes. In order to thermalize the culture plates at 37°C, we used the environmental controller with BioHeater from Asylum Research during all AFM experiments.

### 2.2 AFM nanomechanics

A MFP3D-Bio from Asylum Research was employed in the Force Mapping mode in order to perform morphological and mechanical imaging. A series of single force spectroscopy events (force vs. indentation curve or simply force curves FCs) are acquired regularly spaced on a square matrix while recording topography at maximum force. For all experiments we used spherical micrometric probes (nominal radius R = 5,000 nm) in borosilicate glass attached on a soft cantilever (nominal spring constant k = 0.2 Nm^−1^) from Novascan. Beyond the nominal value, the radius of the spherical probe was characterized by reverse imaging on regular spikes of the TGT1 calibration grid (NT-MDT). Large micrometer-sized probes allowed us to apply reduced local pressure and perform a robust statistical averaging over a mesoscopic interaction area (volume) to better characterize the effect of RTILs on the cell membrane but while also providing a satisfactory lateral resolution compared to the typical cell dimensions. Spring constant and optical lever sensitivity were measured by acquiring a standard force curve on a glass surface in water, successively, using a thermal noise routine from Asylum Research. This experimental setup was successfully employed in recent investigations by the authors (Galluzzi et al., 2018; Tang et al., 2019; Zhang et al., 2022).

Briefly, we selected the parameters for the acquisition of FC: ramp size 8 μm, force setpoint F_MAX_ ≈ 7 nN, approaching velocity v = 32 μm s^−1^, ramp rate 2 Hz. The setpoint force was selected in order to obtain roughly 50% of curve in contact with cell and 50% non-contact considering the higher part of cell. A total of 32 × 32 = 1024 force curves were typically acquired in each force mapping, as we had enough resolution to distinguish single cells in a reasonable scan time (9 min). A total of 10–15 AFM maps can be acquired on the same sample.

The contact point of each FC was individuated by binning the force axis and producing a histogram; the non-contact part is determined as a sharply defined Gaussian distribution, which peaked at zero force. The contact point distance (i.e., the indentation length) was used to correct the morphology map. In fact, morphology is usually obtained at maximum force and indentation maps must be added to retrieve the zero-force morphology, also known as the Morpho channel.

The region of the FC above the width of the Gaussian distribution is considered as the indentation for the fitting procedure. In this framework, the finite thickness correction from Dimitriadis et al. (Dimitriadis et al., 2002) was implemented on standard Hertz model of Eq. 1, considering in the square bracket the height of the cell between probe and substrate:

$$F=\frac{4}{3}\frac{E\sqrt{R}}{\left(1-\nu^{2}\right)}\delta^{3/2}\left[1+1.009\chi_{S}+1.032{\chi_{S}}^{2}+0.578{\chi_{S}}^{3}+0.0048{\chi_{S}}^{4}\right]$$ (1)

where F is the applied force, δ the indentation, ν the Poisson’s ratio, E the effective Young’s Modulus of the cell, and R the radius of the spherical probe and the dimensional parameter $\chi_{S}=\sqrt{R\delta }/h$ . Because there are several differences in the orders of magnitude between probes and cells in Young’s modulus, we always use the effective Young’s Modulus as the modulus of the cell. The finite thickness correction was created while considering a bound layer (i.e., well adherent) and a free-to-move layer with respect to the substrate (Zhou et al., 2020). Since cells are not completely or firmly attached to substrates, we always use a boundary condition as an arithmetic mean of coefficient for bound and not-bound states.

The indentation length depends on the maximum force applied. During data analysis, the percentage of indentation length can be controlled, and mechanical datasets were selected using low indentation (0%–30%) and high indentation (70%–100%). In this work, we defined separate channels for low/high indentation called MechL and MechH, respectively. While MechL is sensitive to the mechanical properties of shallow layers, MechH is more sensitive to deep layers but always mechanically convoluted with MechL.

After reaching the maximum setpoint of force, the probe inverts motion, decreasing applied force and retracting from the sample. The retracting force curve is used to measure the adhesion force necessary to overcome the physicochemical bounds between the probe and cell surface. Adhesion, the so-called Adh channel, was registered as minimum of retracting force curve after zero force alignment of non-contact part. Adhesive interactions are generally small (on average 0.2–0.5 nN v.s. 5–10 nN of indentation force), ensuring the validity of the non-adhesive model in Eq. 1.

### 2.3 Laser scanning confocal microscopy and flowcytometry

RAW 264.7 macrophages were cultured as explained before and then seeded in dishes (35 mm, Ibidi, Gräfelfing, Germany) and 25 cm^2^ flasks for Confocal Laser Scanning Microscopy (CLSM) and Flowcytometry (FLC), respectively. Similar treatment and protocol were used for both techniques. In more detail, cells were fixed with Image-iT Fixative Solution (Invitrogen) for 15 min followed by 0.1% Triton X-100 to improve membrane permeability. Cells were incubated with primary monoclonal antibody Anti -alpha Tubulin Mouse (Servicebio) for 2 h, followed by incubation with a secondary Cy5 conjugated Goat Anti-mouse antibody (Servicebio) for 45 min. Subsequently, cells were incubated with Phalloidin Alexa Fluor 488 (Invitrogen) for 1 h at room temperature.

At this stage of staining, cells were collected by scraping and dispersed in PBS for FLC. A flow cytometer (CytoFLEX S, Backman Coulter) was employed. Actin fibers were visualized by exciting with a 488 nm laser and collecting fluorescent signal between ≈530 nm by the FITC channel. Tubulin microfilaments were detected with a 638 nm excitation laser while detecting ≈670 nm signal. Data were analyzed using FlowJo software, and gating in particular was used to exclude deviant points and doublets.

For CLSM, cells were further incubated with DAPI (Invitrogen) for 15 min and finally cured in ProLong Glass Antifade Mountant before measurements. CLSM (Nikon AXR, Nikon, Japan) used the same excitation/emission paths of FLC, adding 405 nm laser for DAPI excitation and ≈460 nm emission peak. Images were acquired at 1024 × 1024 resolution with four lines averaging, laser power and gain were optimized and maintained constant for all samples. Images were finally analyzed and exported using NIS-Elements AR Analysis (Nikon, Japan).

### 2.4 General pipeline

**FIGURE 1:**
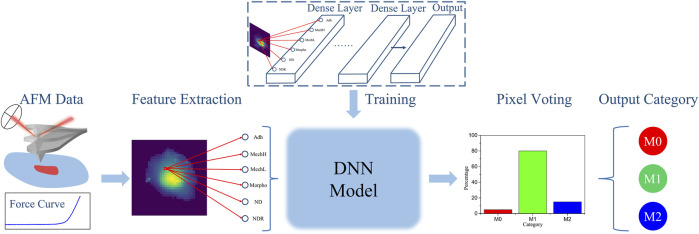
General processing pipeline for the training and prediction process of the DNN model in this work.

**FIGURE 2:**
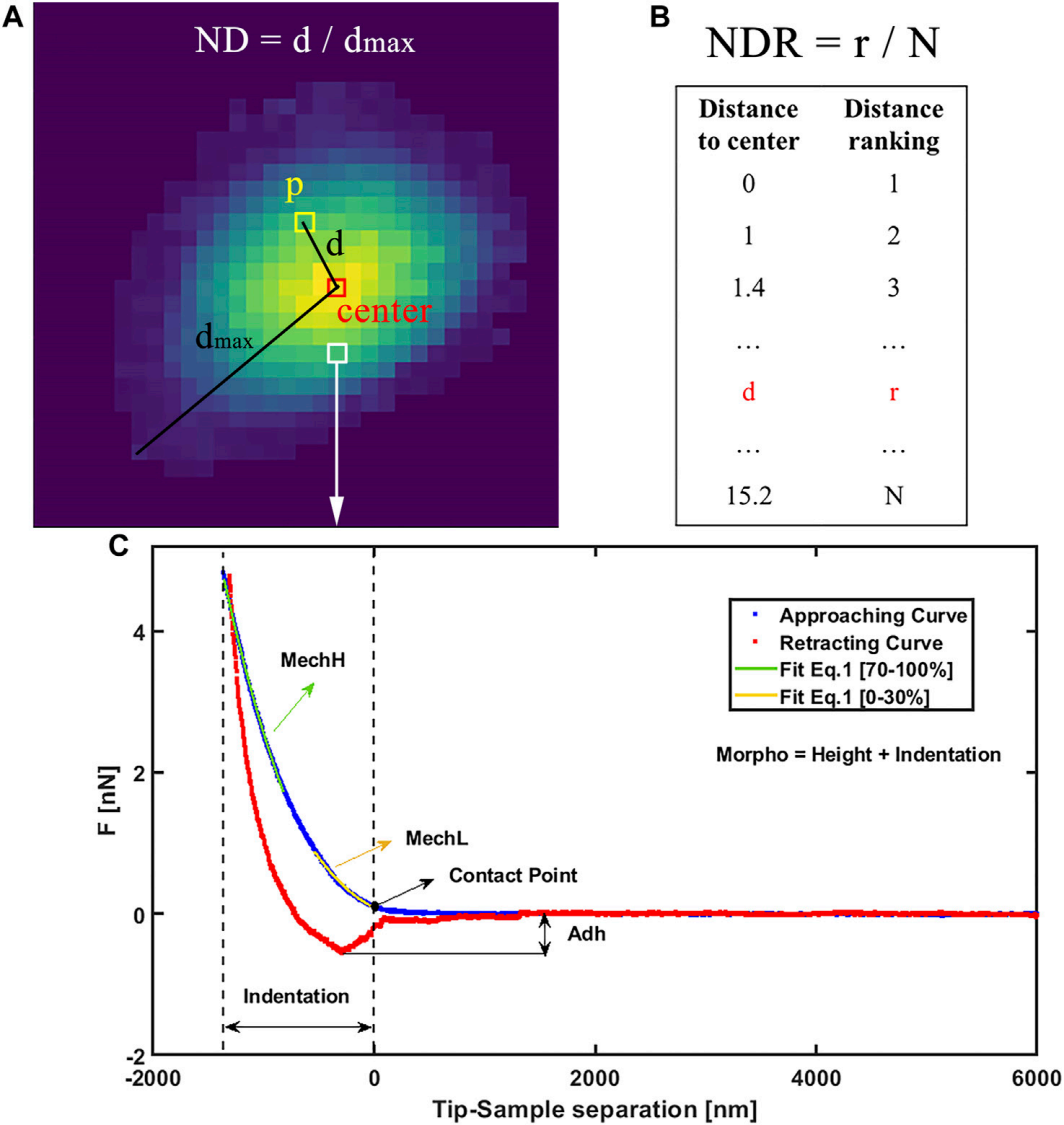
**(A)** Graphical example of ND definition as normalized distance from center and **(B)** definition of NDR as normalized ranking of distance from center. **(C)** AFM experimental force curve on the macrophage, showing approaching (blue) and retracting (red) and contact. On approaching curve MechL is evidenced as result of Eq. 1 fit in range [0%–30%] of indentation and MechH as fit on range [70%–100%]. Adh is evidenced as the force required to detach from the sample surface during the retraction motion. Morpho is calculated as the sum of the height map at maximum force and indentation.

The general processing pipeline of this work is schematized in Figure 1. First, each macrophage is characterized by AFM, and after data analysis, Adh, MechH, MechL, and Morpho channels are obtained as depicted in Figure 2C. Since each pixel represents a single force curve (one indentation event for different locations) of a total force volume, the maps can be divided into pixels with four channels. The transformation from AFM maps to pixels enlarges the dataset, reserves the morphology and mechanical information obtained from the AFM measurement, but drops the location distribution information of each pixel. Therefore, additional distribution information on pixel level is needed in describing the pixels. Besides these four channels, the normalized distance between pixel position and cell center (ND) and the normalized distance ranking between pixel position and cell center (NDR) were also employed for each pixel position of this macrophage to characterize its relative position in this macrophage.

The center position of each macrophage is defined as the average location of all the pixels in it with the weight of height (Morpho value). The ND of a pixel position represents its distance to the center of its macrophage divided by the maximum distance to the center of all pixel positions in this macrophage (shown in Figure 2A). NDR was calculated in the following route (shown in Figure 2B). First, all the pixel positions in a macrophage were sorted from small to large distance to the center. Then these distance rankings were divided by the pixel number the macrophage contains. ND and NDR are important features to maintain useful spatial information when dividing maps into pixels as well as to filter irrelevant information such as some shape details. Although similarly, the relationships between ND and NDR in macrophages and different shapes are clearly different, especially in terms of the roundness of the macrophage (as shown in Supplementary Figure S1). Besides the pixel location information, the relationship between ND and NDR reveals the shape information of a macrophage but from a viewpoint of pixel positions. More importantly, ND and NDR values differ from pixel position to pixel position even in one macrophage, which match well with the other four channels. Since the macrophage maps were divided into pixels to enlarge the dataset, all the data that differ from macrophage to macrophage should be banned, such as information on the shape, size, height, and volume of a macrophage. This kind of macrophage information is invariable when pixel position changes in a macrophage. If such macrophage information is added as additional channels of the pixel positions, the DNN will focus on the macrophage information, ignoring the fluctuation in pixel position information. The large pixel dataset will degenerate into a small macrophage dataset again, with the redundant information of pixel positions, leading to overfitting of the DNN. Therefore, ND and NDR are employed as two additional channels instead of macrophage information. The information of these six channels was fused as data points containing the properties and localization information of each pixel on macrophage’s surface. The phenotype category of this macrophage was used as the training label of the pixel positions in it. Therefore, the four AFM channels of a single cell were expanded in 100-fold data points, each of them containing six features and a label. In this way, the size of the AFM dataset was enlarged by a 10^2^ factor.

After the transformation from picture dataset to list dataset (as shown in Table 1 **)**, a DNN model was trained to obtain the relationship between the features and the labels of pixel positions. The obtained DNN model was used to predict the labels of pixel positions in test dataset, a list dataset with the same structure as the training dataset. The test dataset acts as a blind experiment since the labels of the test dataset were kept unknown both in the DNN test process and the voting process. The category of each test macrophage was predicted by the voting on the result of the pixel positions in it.

**TABLE 1:** Example of a structure of the training set.

Adh	MechH	MechL	Morpho	ND	NDR	Category
0.7249	1.4986	1.9664	4530.2	0.707107	0.688889	M0
0.34208	2.4412	1.8936	1048.2	0.675941	0.875	M2
0.22594	2.8802	2.9136	6842.1	0.166667	0.042254	M1
			⋮			⋮
1.2217	2.0469	1.9219	3940.4	0.316228	0.538462	M2
0.24199	1.8969	2.0261	6175.4	0.517727	0.427083	M0

### 2.5 Data processing

The original AFM datasets selected only whole macrophages or nearly whole macrophages to locate the macrophage center correctly. Then, the AFM data was processed as schematized in Supplementary Figure S2. The AFM dataset was randomly divided into a training set, validation set, and test set by a ratio of 0.6, 0.2, and 0.2, respectively. Then the AFM result of each macrophage in three sets was divided into pixel points and the three picture sets was transformed to three list set as described above. The details of the three sets are shown in Supplementary Table S1. The size of the training set, validation set, and test set are 17729 × 6, 4938 × 6, and 6358 × 6, respectively.

After normalizing all features into [-1, 1], the normalized feature was input into a DNN model.

### 2.6 Model training

The DNN was coded mainly using the TensorFlow deep learning framework (Abadi et al., 2016). As shown in Supplementary Figure S3, the model is built by fully connected layers, with a structure shown in Supplementary Table S2. Adding up all the parameters in Supplementary Table S2, its total trainable parameter number is 3891. The six features of each pixel position were sent into the input layer and propagated forward through seven hidden layers to the output layer to calculate the three classification possibilities. The training set size is 17729 (as shown in Supplementary Table S1), which is enough to train this network. Additionally, dropout, L2 regularization, and early stopping were used in the training process to further reduce the possibility of overfitting. Dropout (Hinton et al., 2012; Srivastava, 2013; Srivastava et al., 2014) was applied to induce part of neurons to stop working with a certain probability, reducing the dependency among the neurons. L2 regularization (Cortes et al., 2009) is used to add the square sum of all the trainable parameters into the objective function (i.e., the error function between the calculated output and the label), reducing the complexity of the DNN fitting function and thus increasing the robustness of DNN. Early stopping (Yao et al., 2007) is an easy way to stop overfitting by stop training when the training effect not getting obviously better within several epochs.

### 2.7 Voting mechanism

The voting result of a macrophage’s phenotype is determined by a weighted voting process of pixel values and positions of cell surfaces. Since the macrophages are adherent, the locations with high thickness are located at the nucleus position, while lower thicknesses represent the periphery, and a different cytoskeletal composition is expected. As the cell height, composition, and heterogeneity change, the relationship between morphology and additional properties may change, so that classification uniqueness and the classification ability of features are influenced. The feature classification ability of different cell composition can be captured as an empirical weight function in which the heterogeneity can be represented by different thickness values over the cell area. Here, a polynomial relation was employed and the voting weight W(x) at pixel position x can be calculated as shown in Eq. 2:

$$W\left(x\right)=\alpha ∙{\left(Morpho\left(x\right)-\beta \left(x\right)\right)}^{\gamma }+C$$ (2)

In which *α* and *γ* are the empirical parameters to represent the importance of the thickness information, while C is another empirical parameter to represent the part independent of the thickness. *Morpho (x)* is the characterized Morpho value at pixel position x. *β(x)* is the estimated Morpho value of the substrate at pixel position x. In this work, since the Morpho value of the substrate is set to 0 in the AFM characterization, *β(x) = 0*. Additionally, the voting weights are set to 1 in order to obtain a universal conclusion without experiential factors, i.e., *α*, *γ* are 0 and C is 1 and thus all weights are equal. More accurate voting results can be expected when changeable weights are used. The changeable weights from Eq. 2 or in another formation can be obtained when the experience of experts is employed, followed by training on an additional validation dataset, which is different from the validation dataset used in DNNs. Supplementary Figure S4 shows how the parameters in Eq. 2 affect the voting results. When the methodology and the pipeline in this work was applied to other cell line, the empirical weight function can be modified and trained reasonably. Additional explanation can be found in the Supplementary Material (Pixel Voting Classification).

### 2.8 Permutation feature importance

To calculate the importance of feature *X*_*i*_, the data of feature *X*_*i*_ in the test set were shuffled as *X*_*i*_’. The new test set obtained was sent into the obtained DNN model for new prediction. The mean absolute error (MAE) between the new prediction and the original prediction was used to characterize the importance of the feature *X*_*i*_ (Fisher et al., 2019). In this work, each of the six features was shuffled 20,000 times, and the normalized average MAE of each feature represents the importance of the feature.

## 3 Results and discussion

### 3.1 AFM mechano-imaging and cytoskeleton analysis

The AFM nanomechanical experiment was performed on RAW 264.7 cells after biochemical stimulation to induce phenotype polarization in comparison with resting phenotype.

**FIGURE 3:**
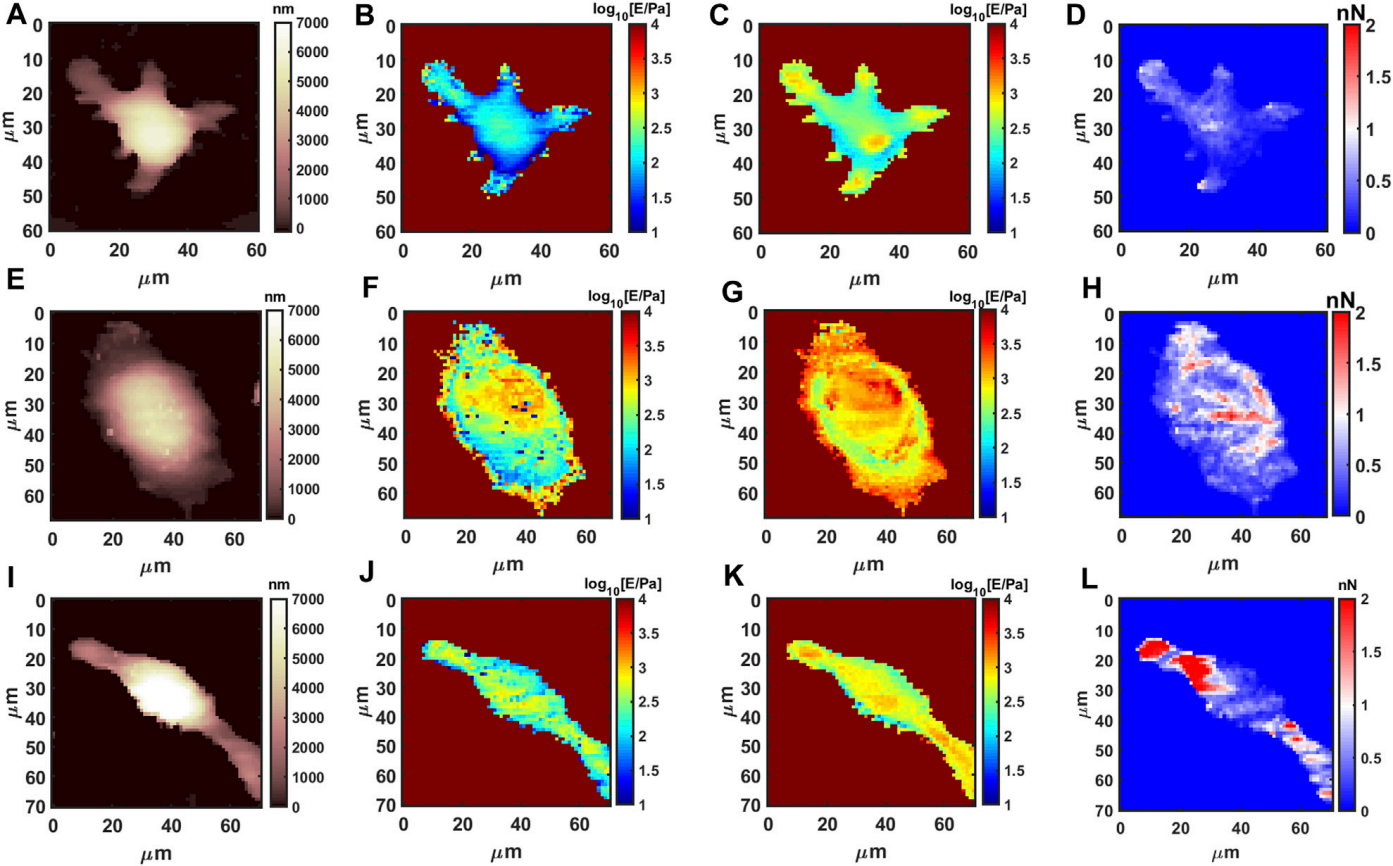
AFM measurements for resting and polarized macrophages. Morphology, shallow Young’s Modulus (MechL), deep Young’s Modulus (MechH) and adhesion maps for RAW 264.7 @37C, respectively, M0 phenotype **(A–D)**, M1 phenotype after 1 μg/mL LPS **(E–H)**, and M2 phenotype after 0.1 μg/mL IL-4 stimulation **(I–L)**.

Live macrophages have a very low Young’s modulus (average 150 Pa for M0 phenotype on full indentation range), which is consistent with the large deformability required during phagocytosis; the Young’s Modulus and adhesion by AFM show significant modifications upon phenotype polarization (M1 or M2). As shown in Figure 3, morphology, the Young’s Modulus and adhesion by AFM are presented using 64 × 64 resolution. For instance, M1 phenotype population shows volume swelling and more rounded and flattened morphology, while the Young’s modulus increases significantly. M2 pro-healing phenotype shows a decrease in cell size, stretched linear morphology, slight increase of the Young’s modulus, and only slight increase in adhesion.

The average values and quantification from the AFM datsets are presented in Supplementary Table S3 and Supplementary Figures S5A–E. Interestingly the quantification using flowcytometry (intensity data in Supplementary Table S4) is in agreeement with AFM; for instance, size and granularity (Supplementary Figures S5F–H) follows the volume measured by AFM (Supplementary Figure S5B). The shallow mechanical modulus (MechL, Supplementary Figure S5C) is relative to the most external layer of cytoskeleton that is in partial agreement with actin signal (Supplementary Figure S5G), while the deep Young’s modulus (MechH, Supplementary Figure S5D) provides information from internal layers, which is in partial agreement with tubulin signal from flowcytometry (FLC) (Supplementary Figure S5H). The partial agreement and mixing is expected because the mechanical modulus of a complex multilayer system is convoluted, for example, high values in shallow layers also contribute when measuring deep layers (like the case of M1). The results show a clear correlation between phenotype determination by biochemical methods and biomechanics, highlighting the feasibility of using AFM mechanical data to distinguish between pro/neuter/anti-inflammatory functional status in macrophages.

Although interesting, the average values from AFM and FLC data do not consider the space distribution of properties. For this reason we compared the AFM analysis with confocal microscopy CLSM. Supplementary Figure S6 shows the overlay of nucleus (blue), actin fibers (green), and tubulin filaments (red) for macrophages under the three conditions. Confocal microscopy, in particular, shows actin is localized at the external layers for all cells and is abundant for central part of M1 cells, while tubulin filaments are mostly near the nucleus or in the elongated filopodia of the M2 phenotype, which is also in agreement with AFM and flowcytometry. While there is no previous information about the nanomechanics of prohealing M2, there is evidence in literature that actin is involved in the M1 phenotype in the re-structuration of shape and cell functionality. Pi et al. (2014) performed AFM on RAW 264.7 upon LPS stimulation, and although cells were fixed, an increase in Young’s modulus, adhesion, and surface roughness were detected. Moreover, they noticed actin redistribution by CLSM, which might be the main reason for the stiffness increase detected by AFM.

### 3.2 CNN model

AFM images corresponding to different channels were used as input in different neural network models for classification. As a compared model, a convolutional neural network (CNN, a deep learning model used on image dataset) was used to classify the phenotypes. The most common data augmentation approach for CNN was to rotate, flip, and crop the AFM data maps of macrophages as shown in Supplementary Figure S7. After that, CNN was trained and the predicted accuracies in confusion matrix are shown in Supplementary Figure S8. However, the performance of the obtained CNN model is a near random guess, which is mainly because of the irregular shape and mechanical heterogeneity of living macrophages. As shown in Supplementary Figure S9 for AFM and Supplementary Figure S6 for CLSM, the shape diversity of macrophages is great both within the same category and between different categories. For a typical small dataset of force volume images on macrophages the performance of CNN is low. The reason is mainly due to the CNN algorithm focusing on spatial details that are not well-defined (due to 32 × 32 resolution) and useless for phenotype discrimination.

### 3.3 DNN model

**FIGURE 4:**
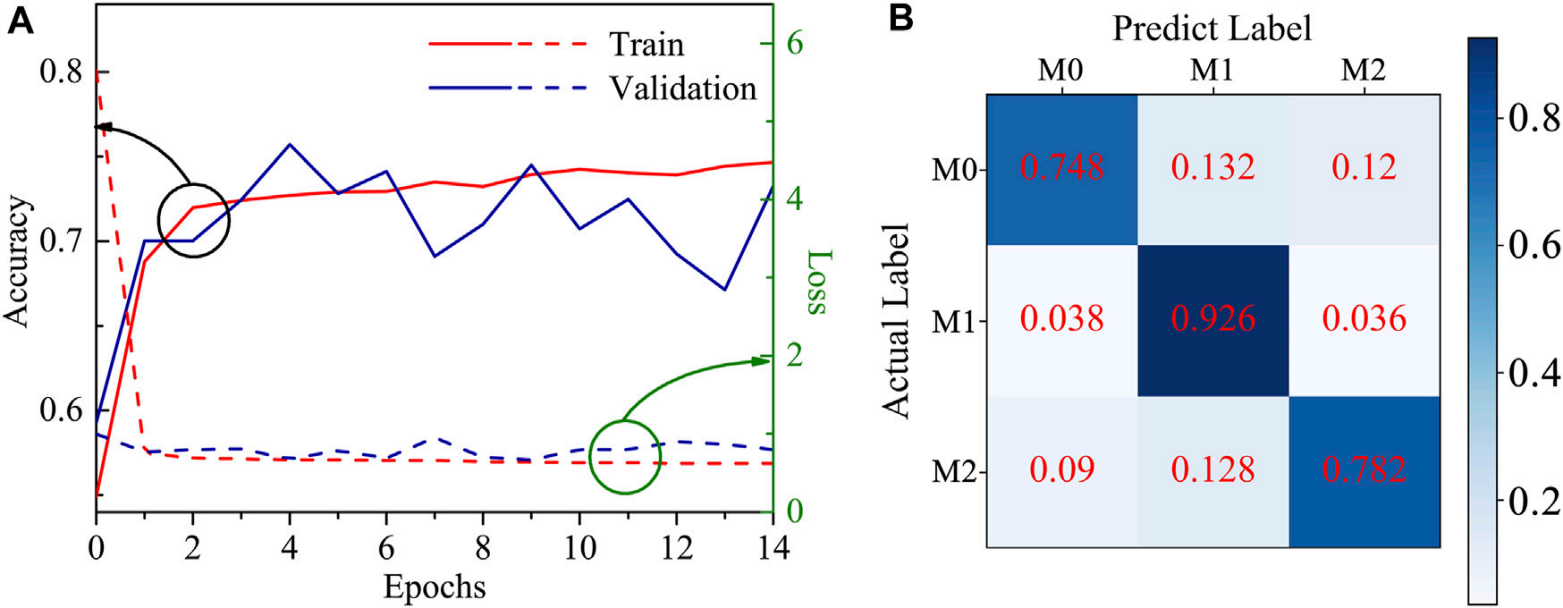
**(A)** The learning curves of the DNN model; **(B)** The predicting accuracy confusion matrix of the DNN model.

The DNN model as mentioned in Section 2.6 was employed, showing better performance. The learning curves in Figure 4A show that the training and validation accuracy of the model reaches 70% after two epochs, indicating a strong learning efficiency. The model prediction results are represented in the confusion matrix of Figure 4B, demonstrating the predicted accuracy of the model for M0, M1, and M2 is 74.8%, 92.6%, and 78.2%, respectively. The results show that the M1 phenotype can be distinguished easily from the other phenotypes, and this is reflected in the fact that mechanical features such as MechL and MechH have higher values compared with M0 and M2. On the otherhand, M0 and M2 have a similar mechanical modulus and similar shapes, causing more mistakes in the phenotype determination.

### 3.4 Voting

According to the category prediction for pixel positions from the DNN model, the category of macrophages was predicted by a voting mechanism. Calculations (shown in Supplementary Figure S10) show that the predicting voting accuracy of a macrophage can be as high as 99.9% if the pixel position prediction accuracy and pixel number of the macrophage are high enough. The calculations assumed all predictions of pixel positions are independent. This is a strong assumption, but it is still useful to understand the reliability and robustness of voting mechanism. For example, in a three-category prediction, the voting predict accuracy of a macrophage can be calculated using Eq. 3:

$$P=C_{m}^{r}∙p_{0}^{r}∙C_{m-r}^{w_{1}}∙p_{1}^{w_{1}}∙C_{m-r-w_{1}}^{w_{2}}∙p_{2}^{w_{2}}∙p_{3}^{m-r-w_{1}-w_{2}}$$ (3)

**FIGURE 5:**
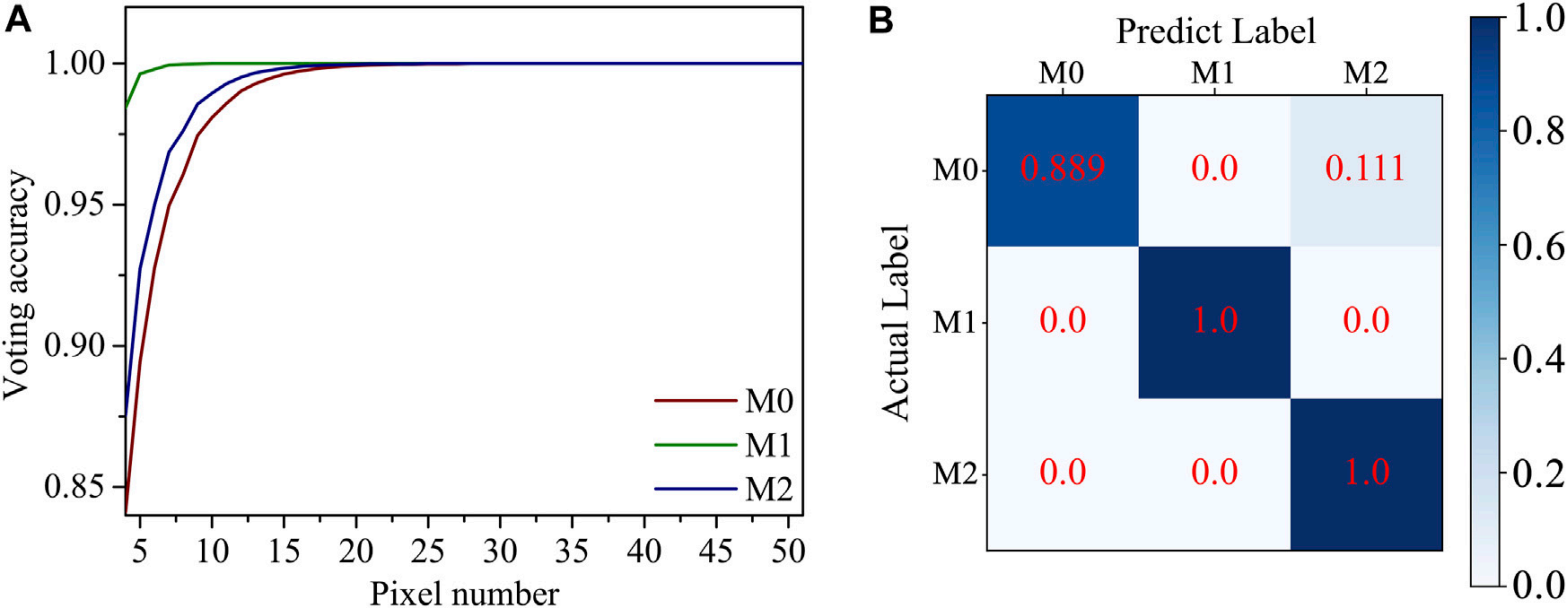
**(A)** The relationship between the theoretical predicting voting accuracy of a macrophage and the pixel number in the macrophage using the pixel predicting accuracy of the trained DNN model. **(B)** The real voting accuracy confusion matrix of macrophage in this work.

In Eq. 3, *P* is the predicting voting accuracy of a macrophage; *m* is the pixel number in the macrophage; *r, w*_*1*_, and *w*_*2*_ are the number of pixel points voting the right category, voting the first wrong category, and voting the second wrong category, respectively; *p*_*0*_, *p*_*1*_, and *p*_*2*_ represent the predicting possibility of pixel points into the right category, the predicting possibility of pixel points into the first wrong category, and the predicting possibility of pixel points into the second wrong category, respectively. The theoretical relationship between the predicting voting accuracy of a macrophage and the pixel number in the macrophage was calculated and is shown in Figure 5A using Eq. 2 and the pixel predicting accuracy of the trained DNN model in Figure 4B.

The theoretical prediction voting accuracy for phenotype increases as the pixel number on the macrophage increases. The theoretical voting accuracies of M0, M1, and M2 macrophages are higher than 99.9% when the pixel number in a macrophage is higher than 20, 7, and 17, respectively. In the real voting process, the predictions of pixel positions are not independent, so the pixel number thresholds for three kinds of macrophages are different. The average pixel numbers of M0, M1, and M2 macrophages in the test set are 104, 334, and 134, respectively, as shown in Supplementary Table S1. The real voting accuracy of macrophages in this work for M0, M1, and M2 macrophages is 88.9%, 100%, and 100%, respectively. As explained in the previous section before applying pixel voting, inflammatory phenotype M1 is clearly well-distinguishable, while the misclassification may come from the similarity between M0 and M2 in modulus and shape.

**FIGURE 6:**
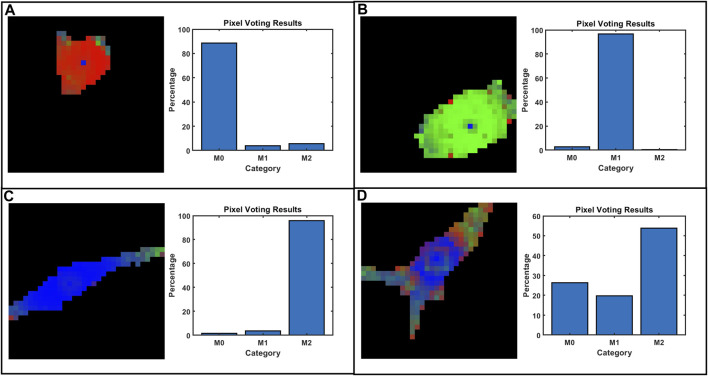
Some representative voting and classification results on selected cells: **(A)** M0, **(B)** M1, **(C)** M2, and **(D)** M2 with poor classification. The pixel predictions of the trained DNN model were artificially colored red for M0, green for M1, and blue for M2.

Typical examples of voting maps are presented in Figure 6 where different colors represent the outcomes of voting classifications pixel by pixel. Figures 6A–C shows representative images of well-classified cells, showing M0 with red, M1 with green, and M2 with blue. Figure 6D represents a poor classification in the M2 group where the modulus of periphery parts is similar to M1 and shape is a hybrid of the three typical conformations. Pixel voting also introduces inherent errors, always discriminating as M2 for the cell center where ND and NDR degenerate to zero (−1 after normalization). Few degenerate points on the cell area are negligible for final classification.

Interestingly, points located at the far edges of macrophage can be mistaken as the M1 phenotype. This is mainly due to the correlation with mechanical moduli (MechL and MechH) increasing at the periphery and being mistaken for the M1 phenotype, which has higher moduli. This is biologically relevant for living macrophages that are concentrating actin cytoskeletons in the periphery during the migration process. Indeed, actin is used as a motor for cell movements, and increasing the density during pulling and traction is also reflected in an increase in the mechanical modulus (Weirich et al., 2021). Movements and an increase in the modulus can shift the evaluation towards the M1 phenotype.

### 3.5 Feature importance analysis

**FIGURE 7:**
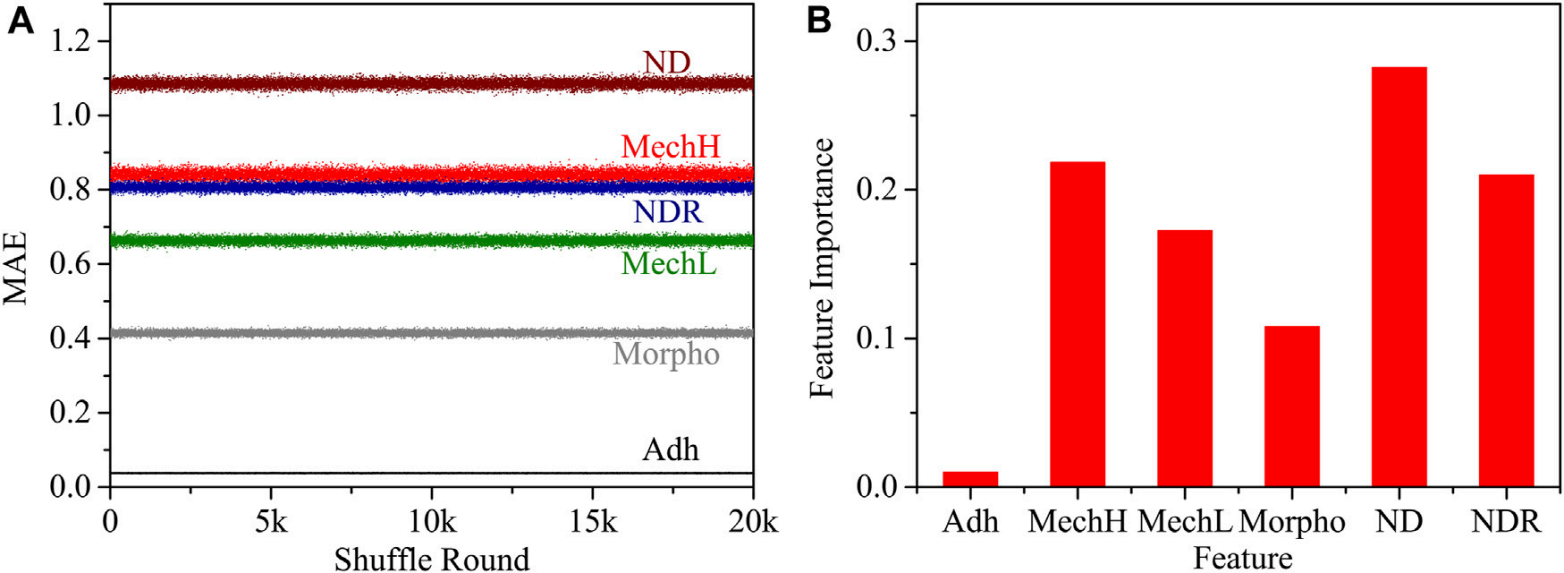
**(A)** The relationship between the MAE of each feature and the shuffle round; **(B)** the normalized feature importance.

The mean absolute error (MAE) induced by changing a feature as mentioned in Section 2.8 was computed as a feature importance analysis. The importance of a feature is calculated by the increase in the model’s prediction error after permuting the feature. A feature is important if shuffling its values increases the MAE obviously. The MAE of each feature and the shuffle round is shown in Figure 7A. The feature importance was calculated as shown in Figure 7B.

In the DNN algorithm, all data layers are employed to classify different phenotypes, and therefore the response of different features is convoluted. It is interesting to notice that the adhesion signal was evaluated as a poor discriminator. Indeed, without functionalization of the spherical probe (silicon oxide) there is no specific difference in membrane adhesion between different phenotypes. Considering all the properties obtained by AFM, the most relevant for classification are mechanical moduli from shallow and deep indentation and their local distribution features ND and NDR, which contain convoluted information from shallow and deep cytoskeleton filaments and position information.

## 4 Conclusion

In this work, a general route to enlarge AFM datasets, train a DNN model by multimodal fusion, and obtain predictions by use of a voting mechanism was proposed. A DNN model was successfully trained on a small AFM dataset of macrophages. The theoretical calculations confirmed the reliability and robustness of voting mechanism, i.e., the prediction voting accuracy of a macrophage, can be as high as 99.9% if the pixel position prediction accuracy and pixel number in the macrophage are high enough. The obtained voting prediction accuracy for M0, M1, and M2 is 88.9%, 100%, and 100%, respectively. This model can be used as a powerful tool to quickly classify macrophages in AFM characterization. The feature importance of the DNN model was calculated, highlighting property distribution and the mechanical modulus as the most performant features for classification. This finding is biologically relevant as flowcytometry and confocal microscopy confirmed that phenotype activation triggers different conformations at the cytoskeleton level. This work not only provides a new approach to train a deep learning model on small AFM datasets, but it also sheds light on deep learning training for other experimental problems hindered by small datasets.

## Data Availability

The raw data supporting the conclusion of this article will be made available by the authors, without undue reservation.

## References

[B1] Abadi M. Barham P. Chen J. Chen Z. Davis A. Dean J. (2016). “Tensorflow: A system for large-scale machine learning,” in This paper is included in the proceedings of the 12th USENIX symposium on operating systems design and implementation (OSDI ’16) (Savannah, GA, USA: OSDI), 16, 265–283.

[B2] Azuri I. Rosenhek-Goldian I. Regev-Rudzki N. Fantner G. Cohen S. R. (2021). The role of convolutional neural networks in scanning probe microscopy: A review. Beilstein J. Nanotechnol. 12, 878–901. 10.3762/bjnano.12.66 34476169 PMC8372315

[B3] Bengio Y. Delalleau O. (2011). “On the expressive power of deep architectures,” in Algorithmic learning theory: 22nd international conference, ALT 2011 (Espoo, Finland: Springer), 18–36.

[B4] Ciasca G. Mazzini A. Sassun T. E. Nardini M. Minelli E. Papi M. (2019). Efficient spatial sampling for AFM-based cancer diagnostics: A comparison between neural networks and conventional data analysis. Condens. Matter 4 (2), 58. 10.3390/condmat4020058

[B5] Cortes C. Mohri M. Rostamizadeh A. (2009). “L2 regularization for learning kernels,” in Uai '09: Proceedings of the twenty-fifth conference on uncertainty in artificial intelligence (AUAI Press), 109–116.

[B6] Cross S. E. Jin Y. S. Rao J. Gimzewski J. K. (2007). Nanomechanical analysis of cells from cancer patients. Nat. Nanotechnol. 2 (12), 780–783. 10.1038/nnano.2007.388 18654431

[B7] Darling E. M. Guilak F. (2008). A neural network model for cell classification based on single-cell biomechanical properties. Tissue Eng. Part A 14 (9), 1507–1515. 10.1089/ten.tea.2008.0180 18620486 PMC2748927

[B8] Dimitriadis E. K. Horkay F. Maresca J. Kachar B. Chadwick R. S. (2002). Determination of elastic moduli of thin layers of soft material using the atomic force microscope. Biophysical J. 82 (5), 2798–2810. 10.1016/s0006-3495(02)75620-8 PMC1302067 11964265

[B9] Fisher A. Rudin C. Dominici F. (2019). All models are wrong, but many are useful: learning a variable's importance by studying an entire class of prediction models simultaneously. J. Mach. Learn. Res. 20 (177), 177–181. https://www.jmlr.org/papers/v20/18-760.html . 34335110 PMC8323609

[B10] Galluzzi M. Schulte C. Milani P. Podesta A. (2018). Imidazolium-based ionic liquids affect morphology and rigidity of living cells: an atomic force microscopy study. Langmuir 34 (41), 12452–12462. 10.1021/acs.langmuir.8b01554 30213187

[B11] Garcia R. (2020). Nanomechanical mapping of soft materials with the atomic force microscope: methods, theory and applications. Chem. Soc. Rev. 49, 5850–5884. 10.1039/d0cs00318b 32662499

[B12] Hinton G. E. Salakhutdinov R. R. (2006). Reducing the dimensionality of data with neural networks. Science 313, 504–507. 10.1126/science.1127647 16873662

[B13] Hinton G. E. Srivastava N. Krizhevsky A. Sutskever I. Salakhutdinov R. R. (2012). Improving neural networks by preventing co-adaptation of feature detectors. arXiv preprint arXiv:1207.0580.

[B14] Leporatti S. Gerth A. Kohler G. Kohlstrunk B. Hauschildt S. Donath E. (2006). Elasticity and adhesion of resting and lipopolysaccharide-stimulated macrophages. FEBS Lett. 580 (2), 450–454. 10.1016/j.febslet.2005.12.037 16376879

[B15] Li M. Xi N. Wang Y. Liu L. (2018). Advances in atomic force microscopy for single-cell analysis. Nano Res. 12 (4), 703–718. 10.1007/s12274-018-2260-0

[B16] Lohrer M. F. Liu Y. Hanna D. M. Wang K. H. Liu F. T. Laurence T. A. (2020). Determination of the maturation status of dendritic cells by applying pattern recognition to high-resolution images. J. Phys. Chem. B 124 (39), 8540–8548. 10.1021/acs.jpcb.0c06437 32881502

[B17] Minelli E. Ciasca G. Sassun T. E. Antonelli M. Palmieri V. Papi M. (2017). A fully-automated neural network analysis of AFM force-distance curves for cancer tissue diagnosis. Appl. Phys. Lett. 111 (14), 143701. 10.1063/1.4996300

[B18] Pi J. Li T. Liu J. Su X. Wang R. Yang F. (2014). Detection of lipopolysaccharide induced inflammatory responses in RAW264.7 macrophages using atomic force microscope. Micron 65, 1–9. 10.1016/j.micron.2014.03.012 25041825

[B19] Rade J. Zhang J. Sarkar S. Krishnamurthy A. Ren J. Sarkar A. (2022). Deep learning for live cell shape detection and automated AFM navigation. Bioengineering 9 (10), 522. 10.3390/bioengineering9100522 36290490 PMC9598706

[B20] Roduit C. Longo G. Benmessaoud I. Volterra A. Saha B. Dietler G. (2012). Stiffness tomography exploration of living and fixed macrophages. J. Mol. Recognit. 25 (5), 241–246. 10.1002/jmr.2184 22528184

[B21] Rotsch C. Braet F. Wisse E. Radmacher M. (1997). AFM imaging and elasticity measurements on living rat liver macrophages. Cell. Biol. Int. 21 (11), 685–696. 10.1006/cbir.1997.0213 9817809

[B22] Sokolov I. Dokukin M. E. Kalaparthi V. Miljkovic M. Wang A. Seigne J. D. (2018). Noninvasive diagnostic imaging using machine-learning analysis of nanoresolution images of cell surfaces: detection of bladder cancer. Proc. Natl. Acad. Sci. U. S. A. 115 (51), 12920–12925. 10.1073/pnas.1816459115 30509988 PMC6304950

[B23] Srivastava N. Hinton G. Krizhevsky A. Sutskever I. Salakhutdinov R. J. T. (2014). Dropout: A simple way to prevent neural networks from overfitting. J. Mach. Learn. Res. 15 (56), 1929–1958. https://www.jmlr.org/papers/v15/srivastava14a.html .

[B24] Srivastava N. (2013). Improving neural networks with dropout. Univ. Tor. 182 (566), 7. http://www.cs.toronto.edu/~nitish/msc_thesis.pdf .

[B25] Suresh S. (2007). Biomechanics and biophysics of cancer cells. Acta Biomater. 3 (4), 413–438. 10.1016/j.actbio.2007.04.002 17540628 PMC2917191

[B26] Tang G. Galluzzi M. Zhang B. Shen Y. L. Stadler F. J. (2019). Biomechanical heterogeneity of living cells: comparison between atomic force microscopy and finite element simulation. Langmuir 35 (23), 7578–7587. 10.1021/acs.langmuir.8b02211 30272980

[B27] Wang H. Zhang H. Da B. Lu D. Tamura R. Goto K. (2021). Mechanomics biomarker for cancer cells unidentifiable through morphology and elastic modulus. Nano Lett. 21 (3), 1538–1545. 10.1021/acs.nanolett.1c00003 33476166

[B28] Weirich K. L. Stam S. Munro E. Gardel M. L. (2021). Actin bundle architecture and mechanics regulate myosin II force generation. Biophysical J. 120 (10), 1957–1970. 10.1016/j.bpj.2021.03.026 PMC8204393 33798565

[B29] Yao Y. Rosasco L. Caponnetto A. (2007). On early stopping in gradient descent learning. Constr. Approx. 26 (2), 289–315. 10.1007/s00365-006-0663-2

[B30] Zhang B. Galluzzi M. Zhou G. Yu H. (2022). A study of macrophage mechanical properties and functional modulation based on the Young's modulus of PLGA-PEG fibers. Biomaterials Sci. 11 (1), 153–161. 10.1039/d2bm01351g 36385648

[B31] Zhou G. Zhang B. Wei L. Zhang H. Galluzzi M. Li J. (2020). Spatially resolved correlation between stiffness increase and actin aggregation around nanofibers internalized in living macrophages. Materials 13 (14), 3235. 10.3390/ma13143235 32708102 PMC7412258

