# Supplementary material for: Deep learning strategy for small dataset from atomic force microscopy mechano-imaging on macrophages phenotypes
Source: Front Bioeng Biotechnol. 2023 Oct 4;11:1259979. doi: 10.3389/fbioe.2023.1259979 (PMC10582561; doi:10.3389/fbioe.2023.1259979)
Supplement: Supplementary file 1 [file DataSheet1.docx]

Supplementary Material

Deep Learning Strategy for Small Dataset from Atomic Force Microscopy Mechano-Imaging on Macrophages Phenotypes

Hao Wu^1^*, Lei Zhang^1^, Banglei Zhao^1^, Wenjie Yang^2^, Massimiliano Galluzzi^2^*

^1^School of Management Science and engineering, Anhui University of Finance & Economics, Bengbu, Anhui, 233030, China.

^2^Shenzhen Institute of Advanced Technology, Chinese Academy of Science, Shenzhen, Guangdong, , China.

*** Correspondence:**Massimiliano Galluzzi [galluzzi@siat.ac.cn](mailto:galluzzi@siat.ac.cn), Hao Wu [120200004@aufe.edu.cn](mailto:120200004@aufe.edu.cn)

**Pixel Voting Classification**

The pixel point voting accuracies of different classification task were simulated using **Eq.S1**-**Eq.S4**, assuming all the pixel points vote independently. *P_2_*, *P_3_*, *P_4_* and *P_5_* is the voting predict accuracy of a macrophage in a two, three, four and five category classification, respectively; *m*, *r*, *w_1_*, *w_2_* and *w_3_* is the pixel number in the macrophage, the number of pixel points voting to the right category, the number of pixel points voting to the first wrong category, the number of pixel points voting to the second wrong category, the number of pixel points voting to the third wrong category, respectively; *p_0_*, *p_1_*, *p_2_*, *p_3_* and *p_4_* represent the predicting possibility of pixel points into the right category, the predicting possibility of pixel points into the first wrong category, the predicting possibility of pixel points into the second wrong category, the predicting possibility of pixel points into the third wrong category and the predicting possibility of pixel points into the fourth wrong category, respectively. Assuming all the wrong predicting possibility are equal in each classification task, the relationship between the pixel point voting accuracies of different classification task, the pixel number in the macrophage and the pixel predict accuracy was shown in **Figure S9**.

$\boldsymbol{P}_{\boldsymbol{2}}\boldsymbol{=}\boldsymbol{C}_{\boldsymbol{m}}^{\boldsymbol{r}}\boldsymbol{\cdot}\boldsymbol{p}_{\boldsymbol{0}}^{\boldsymbol{r}}\boldsymbol{\cdot}\boldsymbol{p}_{\boldsymbol{1}}^{\boldsymbol{w}_{\boldsymbol{1}}}$ (Eq. S1)

$\boldsymbol{P}_{\boldsymbol{3}}\boldsymbol{=}\boldsymbol{C}_{\boldsymbol{m}}^{\boldsymbol{r}}\boldsymbol{\cdot}\boldsymbol{p}_{\boldsymbol{0}}^{\boldsymbol{r}}\boldsymbol{\cdot}\boldsymbol{C}_{\boldsymbol{m-r}}^{\boldsymbol{w}_{\boldsymbol{1}}}\boldsymbol{\cdot}\boldsymbol{p}_{\boldsymbol{1}}^{\boldsymbol{w}_{\boldsymbol{1}}}\boldsymbol{\cdot}\boldsymbol{p}_{\boldsymbol{2}}^{\boldsymbol{m-r-}\boldsymbol{w}_{\boldsymbol{1}}}$ (Eq. S2)

$\boldsymbol{P}_{\boldsymbol{4}}\boldsymbol{=}\boldsymbol{C}_{\boldsymbol{m}}^{\boldsymbol{r}}\boldsymbol{\cdot}\boldsymbol{p}_{\boldsymbol{0}}^{\boldsymbol{r}}\boldsymbol{\cdot}\boldsymbol{C}_{\boldsymbol{m-r}}^{\boldsymbol{w}_{\boldsymbol{1}}}\boldsymbol{\cdot}\boldsymbol{p}_{\boldsymbol{1}}^{\boldsymbol{w}_{\boldsymbol{1}}}\boldsymbol{\cdot}\boldsymbol{C}_{\boldsymbol{m-r-}\boldsymbol{w}_{\boldsymbol{1}}}^{\boldsymbol{w}_{\boldsymbol{2}}}\boldsymbol{\cdot}\boldsymbol{p}_{\boldsymbol{2}}^{\boldsymbol{w}_{\boldsymbol{2}}}\boldsymbol{\cdot}\boldsymbol{p}_{\boldsymbol{3}}^{\boldsymbol{m-r-}\boldsymbol{w}_{\boldsymbol{1}}\boldsymbol{-}\boldsymbol{w}_{\boldsymbol{2}}}$ (Eq. S3)

$\boldsymbol{P}_{\boldsymbol{5}}\boldsymbol{=}\boldsymbol{C}_{\boldsymbol{m}}^{\boldsymbol{r}}\boldsymbol{\cdot}\boldsymbol{p}_{\boldsymbol{0}}^{\boldsymbol{r}}\boldsymbol{\cdot}\boldsymbol{C}_{\boldsymbol{m-r}}^{\boldsymbol{w}_{\boldsymbol{1}}}\boldsymbol{\cdot}\boldsymbol{p}_{\boldsymbol{1}}^{\boldsymbol{w}_{\boldsymbol{1}}}\boldsymbol{\cdot}\boldsymbol{C}_{\boldsymbol{m-r-}\boldsymbol{w}_{\boldsymbol{1}}}^{\boldsymbol{w}_{\boldsymbol{2}}}\boldsymbol{\cdot}\boldsymbol{p}_{\boldsymbol{2}}^{\boldsymbol{w}_{\boldsymbol{2}}}\boldsymbol{\cdot}\boldsymbol{C}_{\boldsymbol{m-r-}\boldsymbol{w}_{\boldsymbol{1}}-\boldsymbol{w}_{\boldsymbol{2}}}^{\boldsymbol{w}_{3}}\boldsymbol{\cdot}\boldsymbol{p}_{\boldsymbol{3}}^{\boldsymbol{w}_{\boldsymbol{3}}}\boldsymbol{\cdot}\boldsymbol{p}_{\boldsymbol{4}}^{\boldsymbol{m-r-}\boldsymbol{w}_{\boldsymbol{1}}\boldsymbol{-}\boldsymbol{w}_{\boldsymbol{2}}-\boldsymbol{w}_{\boldsymbol{3}}}$ (Eq. S4)

**List of Supplementary Figures**


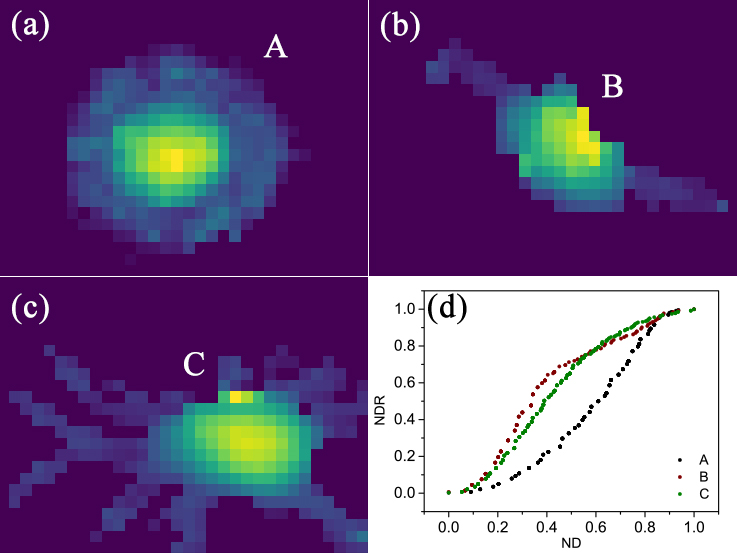


**Figure S1.** (A) – (C) The Morpho pictures of three representative macrophage morphology; (D) the relationship between ND, NDR of pixel points in the macrophage of (A), (B) and (C).


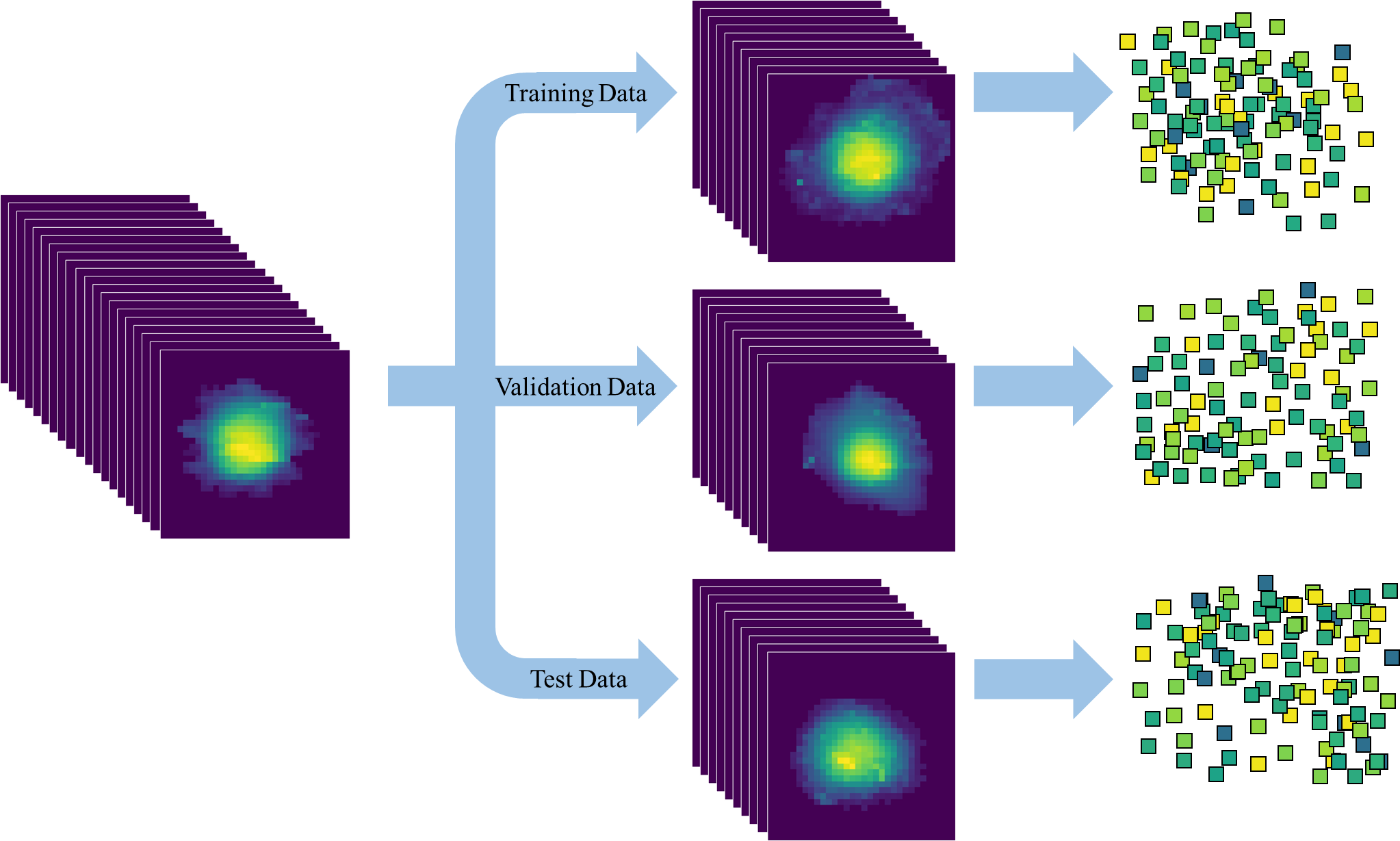


**Figure S2.** Schematic diagram of AFM data processing.


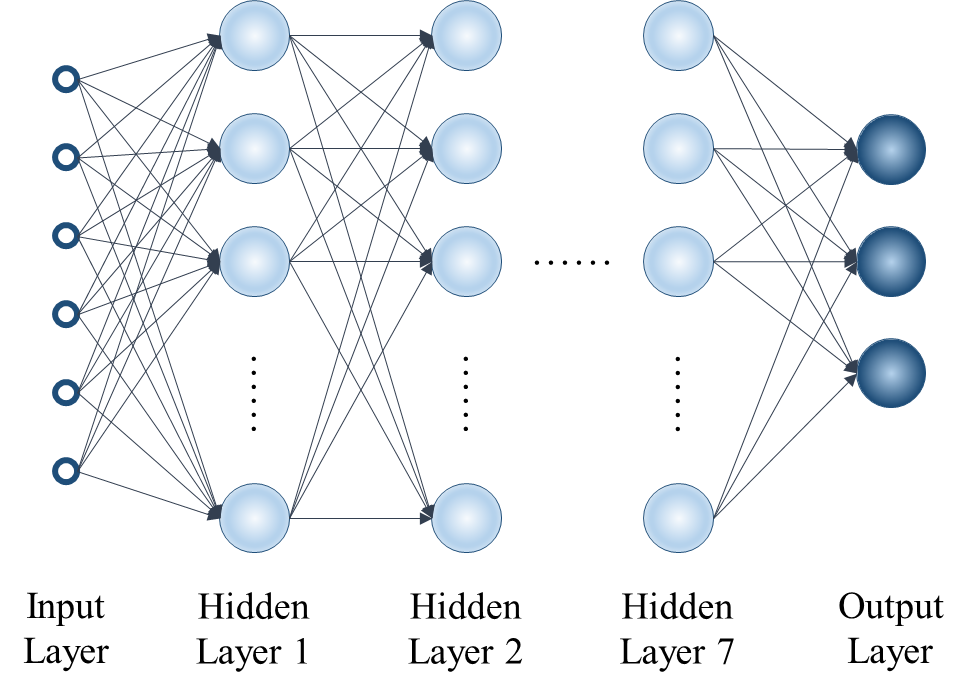


**Figure S3.** The schematic structure of DNN.


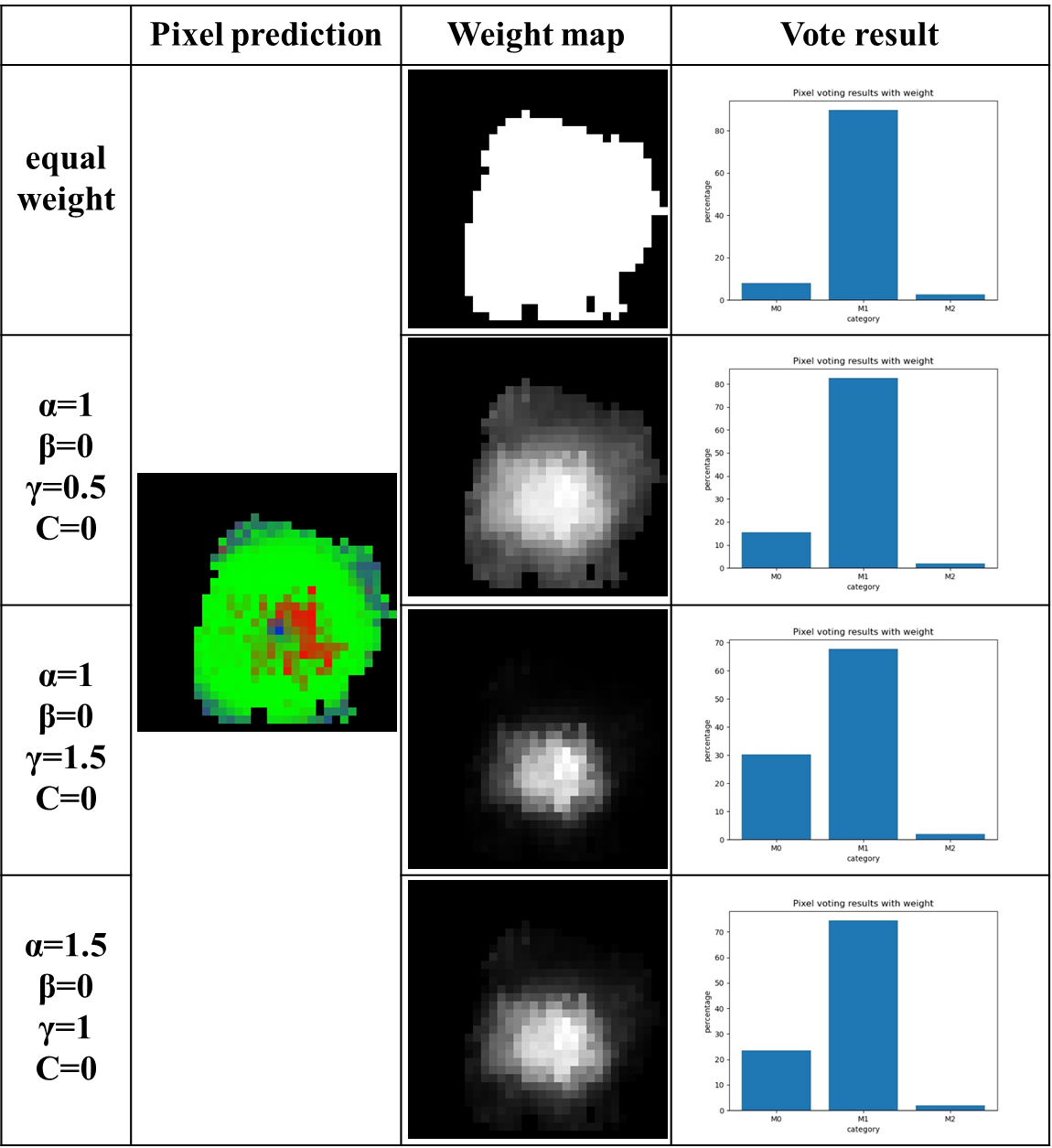


**Figure S4.** The pixel vote results on the same macrophage using the weights calculated with different parameters in Eq. 2.


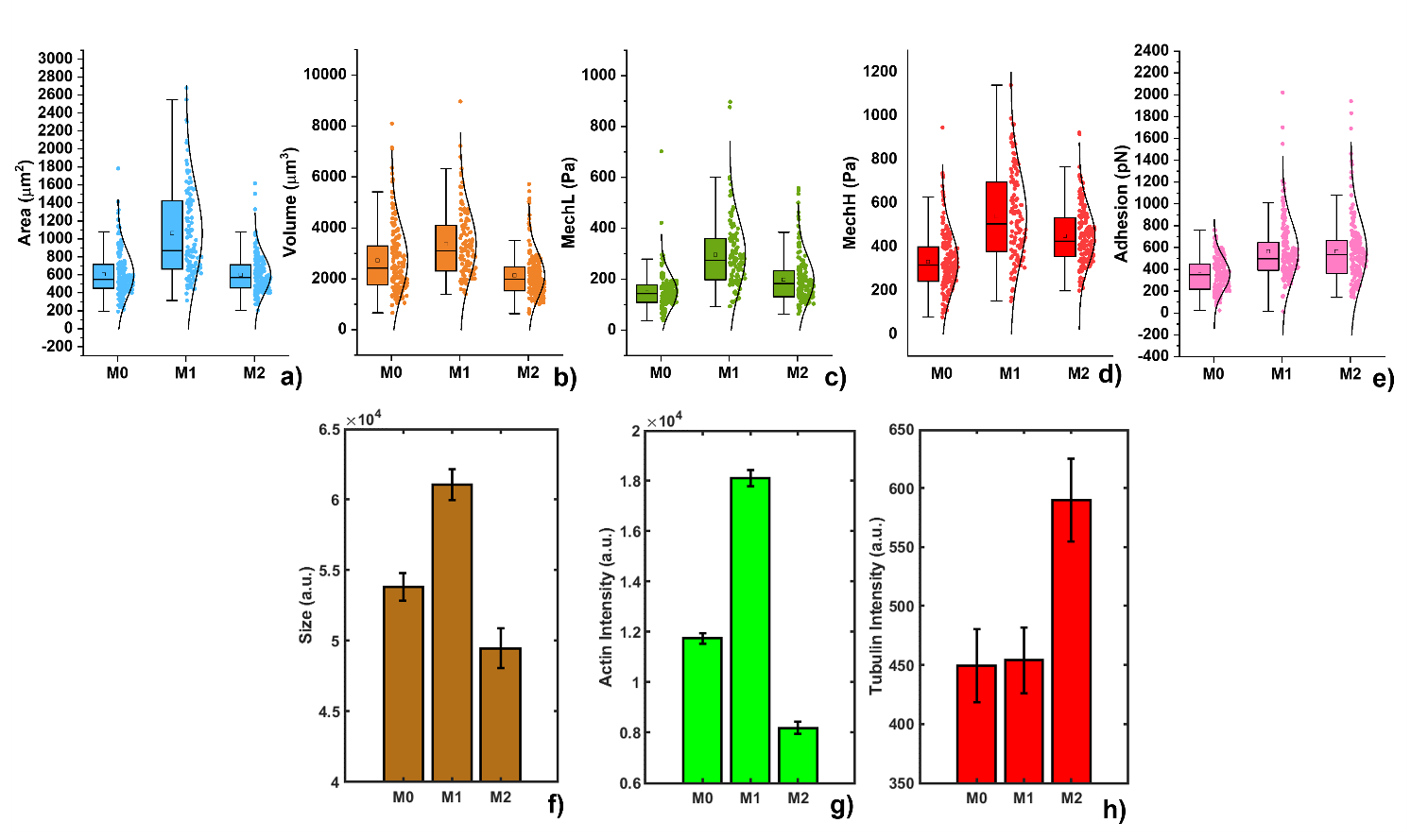


**Figure S5.** AFM and flowcytometry results (average values and errors) for macrophage phenotypes M0, M1 and M2. a) Area from AFM, b) Volume from AFM, c) Shallow Young’s modulus [0-30%] (MechL), d) Deep Young’s modulus [70-100%] (MechH),e) Adhesion force (Adh), f) size from FLC, g) actin signa intensity from FLC, h) tubulin signal intensity from FLC.


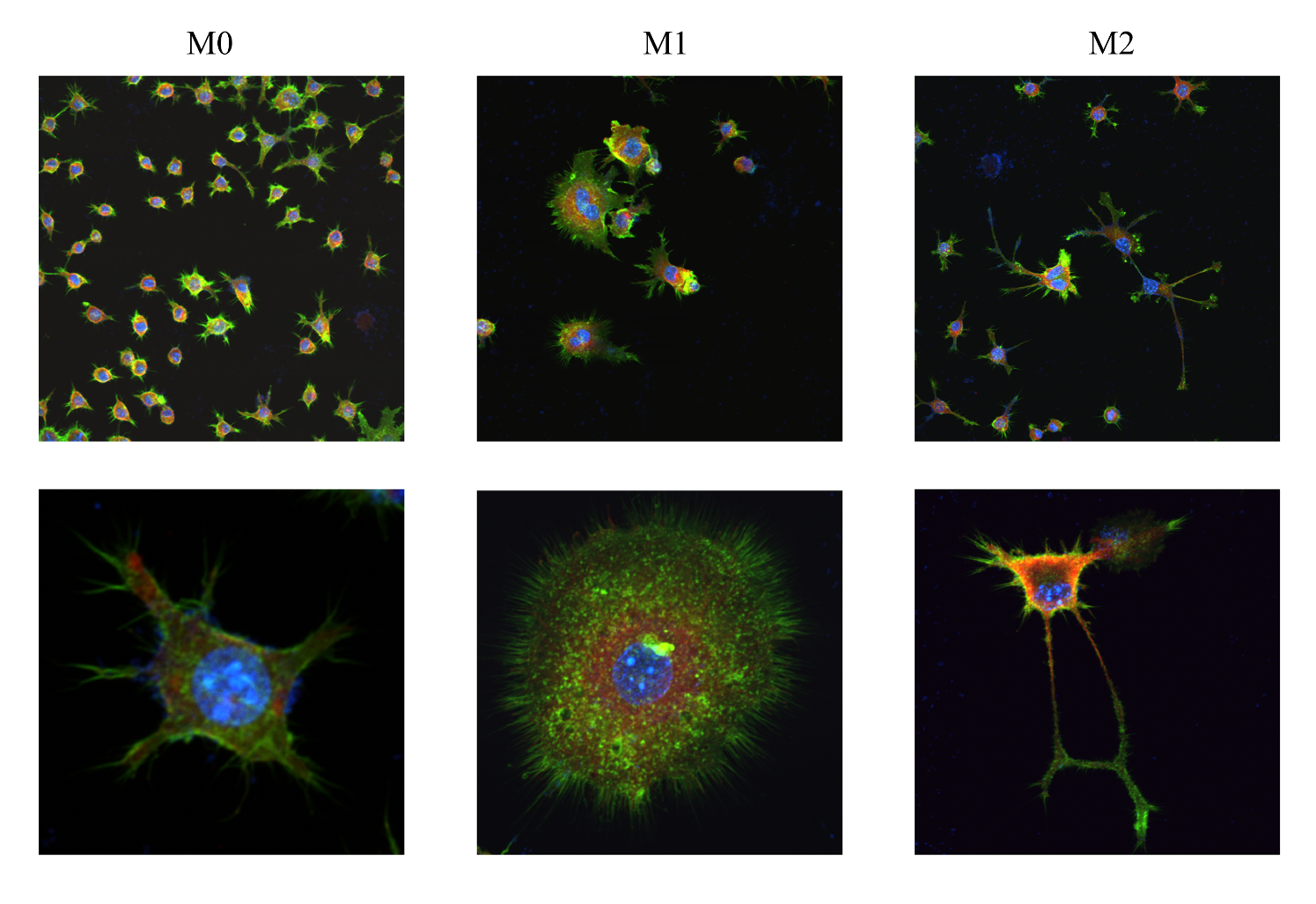


**Figure S6.** CLSM images of fixed RAW 264.7 in 3 different phenotypes M0, M1 and M2. Staining was performed with DAPI (blue) and Rhodamine Phalloidin (green) and alpha-tubulin (red) after fixation and membrane lysis. Top line is a large scale view, while bottom is a zoom selected on a typical cell.

#
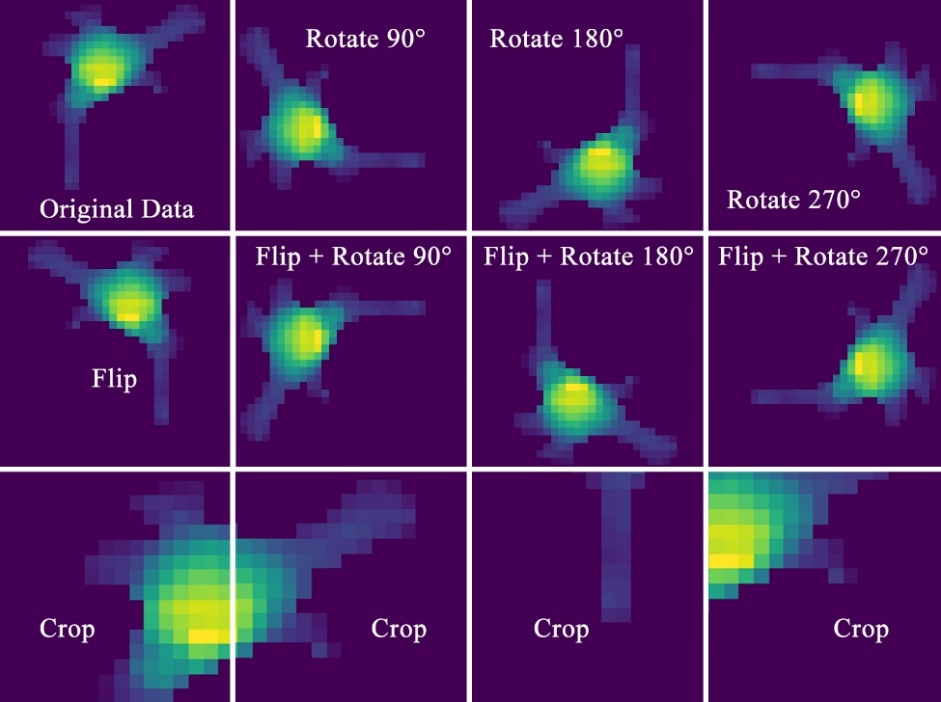


**Figure S7.** Examples of data augmentation. Each one of the original pictures was rotated, flipped, and cropped to obtain 11 new pictures. Thus, the dataset results enlarged and can be used in a typical CNN.


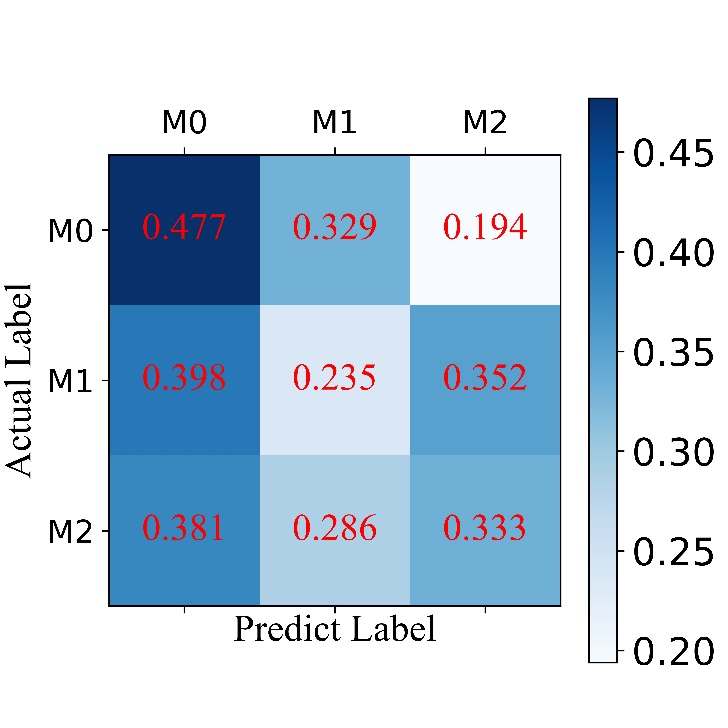


**Figure S8.** Predict confusion matrix of the CNN model trained on the augmented data.

**Figure S9.** Examples of the diversity of macrophages.


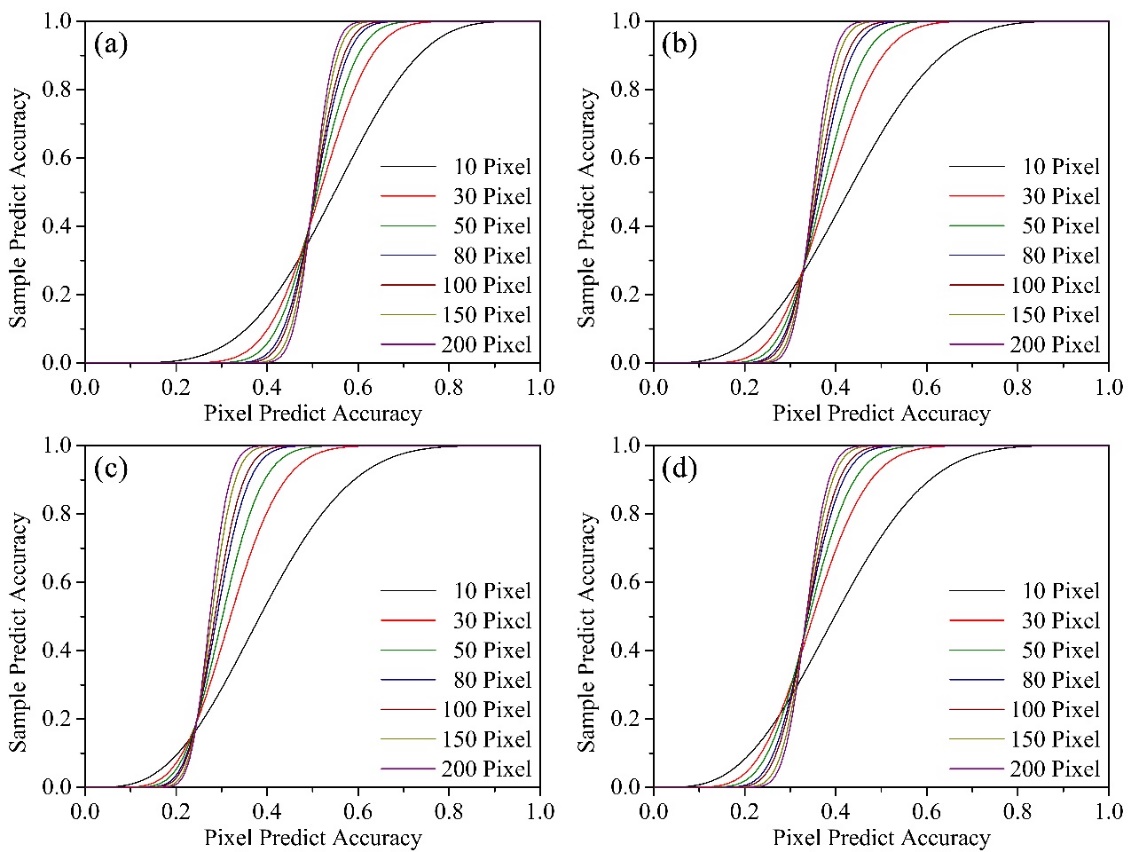


**Figure S10.** The relationship between the pixel position number in a macrophage, pixel predict accuracy and the voting accuracy of a macrophage in a (a) two categories classification; (b) three categories classification; (c) four categories classification; (d) five categories classification.

**List of Supplementary Tables**

| **Train Set** | **M0** | **M1** | **M2** | **Total** |
| --- | --- | --- | --- | --- |
| macrophage | 27 | 34 | 39 | 100 |
| Point | 3589 | 9054 | 5086 | 17729 |
| **Validation Set** | **M0** | **M1** | **M2** | **Total** |
| macrophage | 9 | 11 | 13 | 33 |
| Point | 967 | 2299 | 1672 | 4938 |
| **Test Set** | **M0** | **M1** | **M2** | **Total** |
| macrophage | 9 | 11 | 13 | 33 |
| Point | 937 | 3674 | 1747 | 6358 |

**Table S1.** The details of train set, validation set and test set.

| **Layer Name** | **Layer Type** | **Neuron Number** | **Parameter Number** |
| --- | --- | --- | --- |
| Hidden Layer 1 | Dense | 16 | 112 |
| Hidden Layer 2 | Dense | 16 | 272 |
| Hidden Layer 3 | Dense | 32 | 544 |
| Hidden Layer 4 | Dense | 32 | 1056 |
| Hidden Layer 5 | Dense | 32 | 1056 |
| Hidden Layer 6 | Dense | 16 | 528 |
| Hidden Layer 7 | Dense | 16 | 272 |
| Output Layer | Dense | 3 | 51 |

**Table S2.** The structure of the DNN model used in this work.

|  | **M0 (N=175)** | | **M1 (N=118)** | | **M2 (N=189)** | |
| --- | --- | --- | --- | --- | --- | --- |
|  | mean | std | mean | std | mean | std |
| Area (μm^2^) | 515 | 84 | 793 | 290 | 533 | 70 |
| Volume (μm^3^) | 2106 | 419 | 2697 | 745 | 1873 | 313 |
| MechL (Pa) | 139 | 25 | 277 | 42 | 165 | 31 |
| MechH (Pa) | 308 | 52 | 439 | 75 | 425 | 65 |
| Adh (pN) | 328 | 179 | 554 | 211 | 462 | 156 |

**Table S3.** Data collection from AFM mechano-imaging showing average values (mean) and standard deviation (std) of Area, Volume, shallow Young’s modulus (MechL), deep Young’s modulus (MechH) and adhesion (Adh) for RAW 264.7 resting (M0), after LPS (M1) and after IL4 (M2). Data are additionally depicted in Figure S4(a-e).

|  | **M0 (N=6894)** | | **M1 (N=5410)** | | **M2 (N=5275)** | |
| --- | --- | --- | --- | --- | --- | --- |
|  | mean | std_m | mean | std_m | mean | std_m |
| Actin (a.u.) | 11746 | 212 | 18115 | 321 | 8189 | 234 |
| Tubulin (a.u.) | 449.5 | 31.5 | 454 | 28.2 | 590 | 35 |
| Size (a.u.) | 53824 | 971 | 61056 | 1081 | 49472 | 1413 |

**Table S4.** Data collection from FLC showing average values (mean) and standard deviation of the mean (std_m) of Actin intensity, Tubulin intensity and size for RAW 264.7 resting (M0), after LPS (M1) and after IL4 (M2). Data are additionally depicted in Figure S4(f-h).
